# An Adenovirus Vector Incorporating Carbohydrate Binding Domains Utilizes Glycans for Gene Transfer

**DOI:** 10.1371/journal.pone.0055533

**Published:** 2013-02-01

**Authors:** Julius W. Kim, Joel N. Glasgow, Masaharu Nakayama, Ferhat Ak, Hideyo Ugai, David T. Curiel

**Affiliations:** 1 Cancer Biology Division, School of Medicine, Washington University in St. Louis, St. Louis, Missouri, United States of America; 2 Biologic Therapeutics Center, Department of Radiation Oncology, School of Medicine, Washington University in St. Louis, St. Louis, Missouri, United States of America; 3 Department of Pharmacy, Faculty of Mathematics and Natural Science, University of Groningen, Groningen, The Netherlands; 4 Division of Molecular and Clinical Genetics, Medical Institution of Bioregulation, Kyushu University, Fukuoka, Japan; 5 Department of Microbiology, University of Alabama at Birmingham, Birmingham, Alabama, United States of America; Meharry Medical College, United States of America

## Abstract

**Background:**

Vectors based on human adenovirus serotype 5 (HAdV-5) continue to show promise as delivery vehicles for cancer gene therapy. Nevertheless, it has become clear that therapeutic benefit is directly linked to tumor-specific vector localization, highlighting the need for tumor-targeted gene delivery. Aberrant glycosylation of cell surface glycoproteins and glycolipids is a central feature of malignant transformation, and tumor-associated glycoforms are recognized as cancer biomarkers. On this basis, we hypothesized that cancer-specific cell-surface glycans could be the basis of a novel paradigm in HAdV-5-based vector targeting.

**Methodology/Principal Findings:**

As a first step toward this goal, we constructed a novel HAdV-5 vector encoding a unique chimeric fiber protein that contains the tandem carbohydrate binding domains of the fiber protein of the NADC-1 strain of porcine adenovirus type 4 (PAdV-4). This glycan-targeted vector displays augmented CAR-independent gene transfer in cells with low CAR expression. Further, we show that gene transfer is markedly decreased in cells with genetic glycosylation defects and by inhibitors of glycosylation in normal cells.

**Conclusions/Significance:**

These data provide the initial proof-of-concept for HAdV-5 vector-mediated gene delivery based on the presence of cell-surface carbohydrates. Further development of this new targeting paradigm could provide targeted gene delivery based on vector recognition of disease-specific glycan biomarkers.

## Introduction

Vectors based on human adenovirus type 5 (HAdV-5) have shown considerable utility as gene delivery vectors, particularly in the contexts of vaccination and cancer gene therapy. Innate biological advantages of replication-defective HAdV-5 vectors include *in vivo* stability, highly efficient transfer to both dividing and non-dividing cells and low pathogenicity in humans. In addition, parameters for production of clinical grade Ad vectors are well established. Indeed, as of 2012, roughly one-fourth of gene therapy clinical trials worldwide (428 of 1,843) have employed HAdV-5-based vectors, with two-thirds of all gene therapy trials being for cancer (1,186 of 1,843) [1], [2], [3]. Nonetheless, a limiting feature of HAdV-5-based vectors is that some clinically relevant tissues are poorly transduced due to paucity of the primary receptor molecule for HAdV-5, the coxsackie and adenovirus receptor (CAR) [4], [5]. HAdV-5 tropism is determined by distinct virus-cell interactions: binding of the virus capsid protein, fiber, to the primary high-affinity HAdV-5 receptor CAR, followed by internalization of the virion via secondary interactions with a variety of cell-surface integrins including αvβ5, avβ3 and others [6], [7], [8]. This CAR-dependent tropism hinders HAdV-5-based cancer gene therapy approaches, as decreases in CAR expression appears to coincide with tumor progression [9]. Indeed, the down regulation or absence of CAR expression has been noted in a variety of tumor types such as ovarian, prostate, lung, breast, glioma, melanoma, head and neck carcinoma, colorectal and others [10], [11], [12], [13]. Clearly, the engineering of CAR-independent HAdV-5-based vectors to recognize tumor-selective cell-surface biomarkers could be of great utility.

Aberrant glycosylation of cell surface glycoproteins and glycolipids is a central feature of malignant transformation which may contribute to cancer progression via alteration of tumor cell adhesion and migration [14], [15], [16]. Moreover, determining discrete differences in glycosylation between normal and cancer cells has recently become a central element in discovery of clinically relevant cancer cell biomarkers [17], [18].

Based on the foregoing, we have begun initial development of a gene delivery strategy wherein HAdV-5 vectors are configured to target cell-surface glycans. As a first step toward this goal, we have developed a unique HAdV-5 vector that contains the head and tandem carbohydrate binding domains from the fiber protein of porcine adenovirus type 4 (PAdV-4) NADC-1 strain [19]. Recently, *in silico* structure prediction and high-resolution crystal structure analyses have shown that the PAdV-4 fiber protein contains tandem carbohydrate binding domains (CBDs) which allow the C-terminus of this fiber to bind to carbohydrate chains containing lactose and *N*-acetyl-lactosamine units [20], [21]. We reasoned that genetic incorporation of the PAdV-4 CBDs into the HAdV-5 virion would be potentially useful as a novel means to direct CAR-independent infection of cells using glycosylated cell surface molecules as primary attachment sites. Here, we report the construction and tropism characterization of a novel glycan-targeted HAdV-5-based vector and confirm the ability of this vector to achieve glycan-mediated gene transfer.

## Materials and Methods

### Cell Lines

Human embryonic kidney (HEK) 293, CHO-Pro5 and CHO-Lec8 cells, human embryonic rabdomyosarcoma RD cells, human breast carcinoma MCF-7 cells, prostate adenocarcinoma PC-3, and ovarian OV-3 cells were obtained from the American Type Culture Collection (ATCC; Manassas, VA). Human pancreatic carcinoma cell lines BxPC-3 and Hs766-T were purchased from Boehringer Ingelheim. The OV-4 ovarian adenocarcinoma cell line (formerly referred to and published as OVCA 433 cells [22], [23] was kindly provided by Dr. Timothy J. Eberlein, Washington University in St. Louis, St Louis, MO. Human ovarian adenocarcinoma SKOV3.ip1 cells were obtained from Janet E. Price (M.D. Anderson Cancer Center, Houston, TX) [24]. Chinese hamster ovary (CHO) cells and CHO-hCAR cells stably expressing human CAR were provided by Jeffrey M. Bergelson [5]. All cell lines were maintained in culture media recommended by each supplier. All media contained 10% fetal bovine serum, (FBS; Hyclone; Logan, UT), 2 mM L-glutamine, 100 U/ml penicillin, and 100 mg/ml streptomycin (Mediatech, Inc., Herndon, VA). All cells were incubated at 37°C in 5% CO_2_ in humidified conditions.

### Plasmid construction

A 1,750-bp region containing the PAdV-4 fiber knob and carbohydrate binding domains (amino acids 121–703) of the fiber protein was amplified from cell lysates containing wild type PAdV-4 virus obtained from the US Department of Agriculture National Veterinary Services Laboratory (Ames, Iowa) using the following primers: (PAd4 knob fwd) 5′-TGTGGACGGGGCCTGCTC-3′ and (PAd4 knob rev) 5′-TTTATTACAGTATCTGAGG-3′. The stop codon (TAA) and poly-adenylation signal (TAAA) are underlined. Plasmid pSHAFT, a cloning vector containing the Ad5 fiber gene with the knob region deleted and replaced by a small linker containing *Sma*I and *Eco*ICRI restriction sites [25], was linearized by *Sma*I and *Eco*ICRI digestion, leaving two blunt ends. Following gel purification, the PAdV-4 knob domain PCR product was ligated into linearized pSHAFT resulting in pSHAFT-PK and positive clones were screened for correct orientation via restriction enzyme digest. This plasmid contains the chimeric fiber gene encoding the complete Ad5 fiber shaft in-frame with the PAdV-4 knob domain. A stop codon and poly-adenylation sequence is present at the 3′ end. The chimeric fiber gene in pSHAFT was digested with *Nco*I and *Mun*I to liberate the DNA fragment containing the carboxy terminus of the HAdV-5 shaft and the PAdV-4 knob domain. This fragment was ligated into the *Nco*I-*Mun*I-digested fiber shuttle vector pNEB.PK.3.6 [25], resulting in pNEB.PK.3.6-PK.

### Generation of recombinant adenovirus

The recombinant Ad5Luc1-PK genome containing the chimeric PAdV-4 fiber gene was derived by homologous recombination in *Escherichia coli (E. coli) strain* BJ5183 with *Swa*I-linearized rescue plasmid pVK700 [26] and the fiber-containing *Pac*I-*Kpn*I-fragment of pNEB.PK.3.6-PK, essentially as described [27]. Plasmid pVK700 is derived from pTG3602 [28], but contains an almost complete deletion of the fiber gene and contains a firefly luciferase reporter gene driven by the cytomegalovirus immediate early promoter in place of the E1 region. The recombinant genome of Ad5GFP1-PK containing the chimeric PAdV-4 fiber gene was derived by homologous recombination in BJ5183 cells with fiber shuttle plasmid pKan3.1-PK which contains the same chimeric fiber gene as pNEB.PK.3.6-PK described above, and *Swa*I-linearized rescue plasmid pVK900 [29]. Plasmid pVK900 is a fiber-deleted HAdV-5 genome plasmid essentially the same as pVK700 except that EGFP is encoded in the E1 region (supplied by Victor Krasnykh, University of Texas MD Anderson Cancer Center). All genomic clones were sequenced and analyzed by PCR prior to transfection of HEK 293 cells. Ad5Luc1 is a replication defective E1-deleted Ad vector containing a firefly luciferase reporter gene driven by a cytomegalovirus promoter [30]. All vectors were propagated on HEK 293 cells and purified by equilibrium centrifugation in CsCl gradients by standard protocols. Viral particle (v.p.) concentration was determined at 260 nm by the method of Maizel *et al.* [31] by using a conversion factor of 1.1×10^12^ viral particles/absorbance unit.

### PCR Analysis of the Fiber Region

Genomic DNA contained in Ad5Luc1, Ad5Luc1-PK and PAdV-4 viral particles was used as templates for PCR amplification of fiber genes using a HAdV-5-specific primer set: (fwd) 5′-CAGCTCCATCTCCTAACTGT-3′ and (rev) 5′-TTCTTGGGCAATGTATGAAA-3′ and a PAdV-4-specific primer set: (fwd) 5′-TGTGGACGGGGCCTGCTC-3′ and (rev) 5′-TTTATTACAGTATCTGAGG-3′. Wild type PAdV-4 virus was used as a positive control.

### Western Blot Analysis

Purified virus particles (5.0×10^9^) were diluted in Laemmli buffer and incubated at room temperature (unboiled samples) or 95°C (boiled samples) for 10 minutes and loaded onto a 4–20% gradient SDS-polyacrylamide gel (Bio-Rad, Hercules, CA). Following electrophoretic separation, Ad capsid proteins were electroblotted onto a PVDF membrane and detected with a 4D2 monoclonal anti-fiber tail primary antibody diluted 1/3,000 (Lab Vision, Freemont CA). Immunoblots were developed by addition of a secondary horseradish peroxidase-conjugated anti-mouse immunoglobulin antibody at a 1/3,000 dilution (Dako Corporation, Carpentaria, CA), followed by incubation with 3-3′-diaminobenzene peroxidase substrate, DAB, (Sigma Chemical Company, St. Louis, MO).

### One-step Growth Analysis of Ad Vectors

HEK 293 cells were grown to 80% confluence in 6 wells with 2 ml of medium containing 2% FBS. They were infected with HAdV-5 vectors at a multiplicity of infection (MOI) of 10 plaque forming units (PFU)/cell. The infected cells and growth media were harvested 12, 24 and 48 hours post-infection. The cells were then lysed by three freeze/thaw cycles. The supernatants were collected following centrifugation of the cell lysates at 4,000×*g* for 10 minutes at 4°C and used for subsequent infection.

### Ad-Mediated Gene Transfer Assays

Cells were plated in 24-well plates and were transduced for 1 hour at 37°C with each Ad vector diluted to 100–300 viral particles/cell in 500 µl of transduction media containing 2% FBS. Following the incubation, virus-containing media was replaced with fresh media containing 2% FBS and cells were maintained at 37°C in an atmosphere containing 5% CO_2_. Cells were harvested 24 hours post-transduction in passive lysis buffer and gene transfer was determined using a luciferase activity assay system (Promega, Madison, WI). Fluorescence microscopy was performed with an inverted IX-70 microscope (Olympus) using a 20× objective. Cells were infected with Ad5GFP1-PK for 24 hours prior to imaging.

For experiments assessing the competitive inhibition containing of vector binding to cells, recombinant fiber knob protein [32] at 0.5, 5.0 and 50 µg/ml final concentration or recombinant PAdV-4 carbohydrate binding domain (CBD) protein [20] at 0.5, 5.0, 50 and 100 µg/ml was incubated with various cell lines at 37°C in media containing 2% FBS for 20 minutes prior to the addition of HAdV-5 vectors. Following transduction with Ad vectors, cells were rinsed with media to remove unbound virus and blocking proteins, and were maintained at 37°C in an atmosphere containing 5% CO_2_.

To inhibit glycosylation of cellular proteins, chemical inhibitors of glycosylation were used. CHO-Pro5 cells were incubated with medium containing 10 µg/ml swainsonine (Sigma, Saint Louis, MO., S8195) and/or 1 µg/ml benzyl-α-GalNAc (Sigma B4894) for 24 hr at 37°C, followed by addition of Ad vectors in media containing 2% FBS.

### Biodistribution of Gene Expression

Female C57BL/6 mice (Charles River Laboratories, Wilmington, MA), aged 6–8 weeks were injected intravenously through the lateral tail vein with 1×10^11^ VP of Ad5Luc1 or Ad5Luc1-PK in 100 µl of PBS. After 48 hours mice were sacrificed and livers, lungs, spleens, hearts and kidneys were harvested and representative sections were frozen in liquid nitrogen immediately. The frozen organ samples were homogenized with a Mini Beadbeater (BioSpec Products, Inc., Bartlesville, OK) in 2 ml micro-tubes (Research Product International Corp., Mt. Prospect, IL) within 100 µl of 1.0 mm zirconia/silica beads (BioSpec Products, Inc.) and 1 ml of Cell Culture Lysis Buffer (Promega), then centrifuged at 14,000 rpm in a tabletop microfuge for 2 min. Luciferase activity was measured as above. Mean background luciferase activity was subtracted. All luciferase activities were normalized by protein concentration in the tissue lysates. Protein concentrations were determined using a Bio-Rad DC protein assay kit (Bio-Rad, Hercules, CA). Mice were kept under pathogen-free conditions according to the American Association for Accreditation of Laboratory Animal Care guidelines. Animal protocols were reviewed and approved by the UAB Institutional Animal Care and Use Committee.

## Results

### Generation of a fiber-modified HAdV-5 vector containing the PAdV-4 knob and carbohydrate binding domains

**Figure 1:**
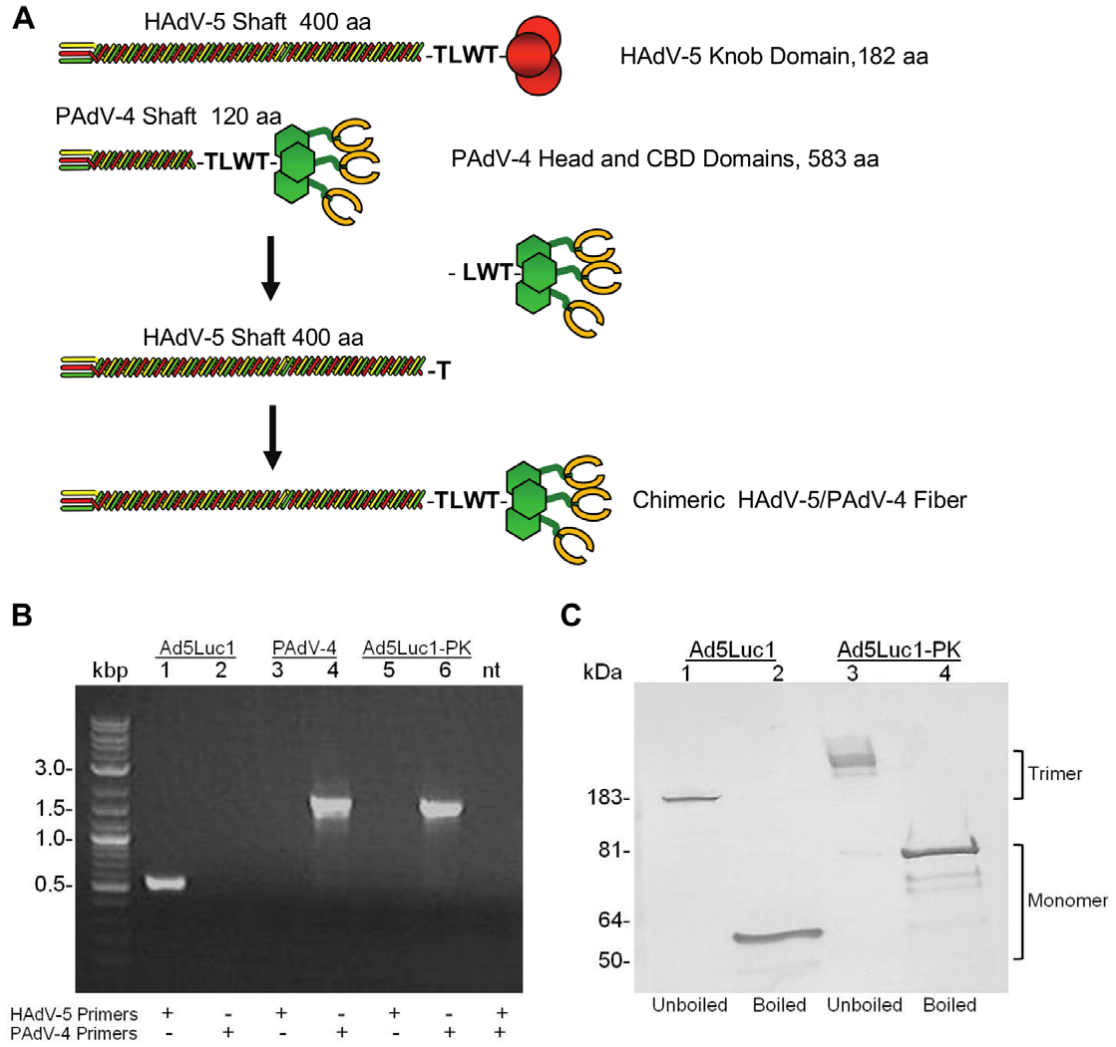
Diagram depicting the design of the Ad5Luc1-PK chimeric fiber and molecular validation of Ad5Luc1-PK virus particles. (A) Schematic diagram of the wild type HAdV-5 and PAdV-4 fiber proteins and the construction of the chimeric fiber of Ad5Luc1-PK and Ad5GFP1-PK. The HAdV-5 fiber knob domain (residues 400 to 582) was replaced with residues 120 to 703 of the fiber protein of PAdV-4, NADC-1 strain. The PAdV-4 fiber head and flexible domains (residues 120–392) are shown in green and the two tandem CBDs (shown in yellow) are located between residues 393–681 of the PAdV-4 fiber sequence [20]. The T-L-W-T peptide sequence joining the shaft and knob domains of both fibers is shown in bold. (B) PCR analysis of fiber genes in the Ad genomes using viral particles as PCR templates. Purified Ad5Luc1 virus particles (lanes 1 and 2), cell lysate containing wild type PAdV-4 virus particles (lanes 3 and 4) and purified Ad5Luc1-PK virus particles (Lanes 5 and 6) were used as DNA templates to amplify the knob domain of each fiber gene with a HAdV-5-specific primer set (lanes 1, 3, 5 and 7) or with PAdV-4-specific primers (lanes 2, 4, 6 and 7) resulting in 530 bp or 1750 bp products, respectively. See Methods for PCR primer sequences. PAdV-4 is the wild type virus and was used as a control. Lanes containing DNA size standards (kbp) and no PCR template (nt) are designated. (C) Western blot analysis of fiber proteins from purified virus particles. 5×10^9^ v.p. of Ad5Luc1 with wild type Ad5 fiber (lanes 1 and 2) or Ad5Luc1-PK with chimeric fiber (lanes 3 and 4) were suspended in Laemmli buffer prior to SDS-PAGE and western blotting analysis with a mAb directed against the HAdV-5 fiber tail domain. Samples marked “Boiled” in lanes 2 and 4 were heat denatured at 95°C prior to electrophoresis. Fiber monomers and trimers are indicated. Molecular mass markers indicate kiloDaltons (kDa).

The fiber protein of PAdV-4 NADC-1 is comprised of a homotrimer of 703 amino acids (Fig. 1A). Predicted functional domains include a tail domain (residues 1–37) containing a penton interaction sequence, a short shaft domain (residues 38–120) with six predicted triple beta-spiral repeats [33] and a fiber head domain homologous to other Ad fiber knob domains (residues 121–287) [20]. This fiber also contains a unique C-terminal domain composed of two tandem CBDs (residues 393–681) that bind carbohydrates containing lactose and *N*-acetyl-lactosamine units [21]. Almost all mastadenoviruses contain a conserved threonine-leucine-tryptophan-threonine (TLWT) motif at the N-terminus of the fiber knob domain, and in human Ad2 and Ad5 a flexible region separating the shaft and the knob domains precedes this motif [34]. We exploited this modular fiber structure to substitute the coding region of the PAdV-4 knob and CBD domains for the HAdV-5 fiber knob sequence while retaining the TLWT motif common to both fibers [35]. We constructed a recombinant E1-deleted HAdV-5 genome (Ad5Luc1-PK) containing the chimeric HAdV-5 shaft/PAdV-4 fiber gene and a firefly luciferase reporter gene controlled by the CMV immediate early promoter/enhancer. The Ad5Luc1-PK vector was rescued following transfection of HEK 293 cells and large-scale preparations of Ad5Luc1-PK and the Ad5Luc1 control vector were produced and purified by double CsCl gradient centrifugation. Ad5Luc1-PK viral particle concentration in full preparations ranged from 1.2×10^11^ to 1.25×10^12^ v.p./ml, similar to that of the Ad5Luc1 control vector containing the HAdV-5 wild type fiber. The Ad5Luc1 vector is isogenic to Ad5Luc1-PK except for the fiber locus.

We confirmed the fiber genotypes of Ad5Luc1 and Ad5Luc1-PK vectors via diagnostic PCR using primer pairs specific for the fiber knob domain and genomes from purified virus particles as PCR templates. Genomic DNA from wild type PAdV-4 was used as a positive control. We observed the expected PCR products for the wild type HAdV-5 fiber knob domain (530 bp) and the PAdV-4 fiber knob and CBD domains (1,750 bp) (Fig. 1B).

We performed SDS-PAGE followed by western blot analysis on purified viral particles to verify that Ad5Luc1-PK contains correctly trimerized chimeric fiber proteins (Fig. 1C). Blots were probed with a monoclonal primary antibody (4D2) directed against the fiber tail domain common to both HAdV-5 and chimeric fiber molecules. In samples that were not heat denatured (Fig. 1C, unboiled) we observed bands at 183 kDa and an estimated 250 kDa, corresponding to trimers of the HAdV-5 fiber and chimeric fibers, respectively. Further, bands in boiled samples resolved at apparent molecular masses of 60 kDa for the wild type HAdV-5 fiber and 90 kDa for the chimeric fiber in Ad5Luc1-PK, representing fiber monomers.

### Ad5Luc1-PK viral replication and thermostability

**Figure 2:**
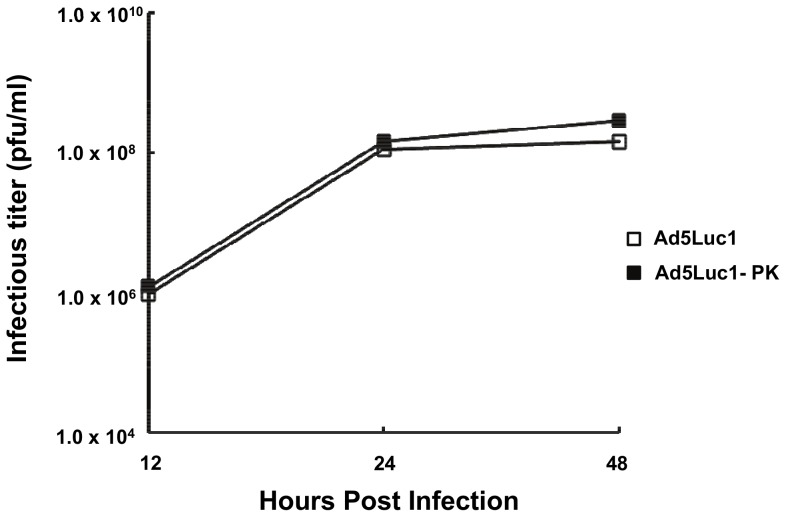
Comparison of viral replication kinetics. HEK 293 cells were infected with Ad5Luc1 (open squares) or Ad5Luc1-PK (filled squares) at an MOI of 10 PFU/cell for various times. Infectious titers were determined by the TCID_50_ method. Each data point represents an average of triplicates.

We next characterized the viral replication of Ad5-Luc1-PK by one-step growth curve analysis in order to identify any growth defect arising from the incorporation of the chimeric fiber protein [36], [37]. As shown in Figure 2, the kinetics of Ad5Luc1-PK replication in HEK 293 cells observed at 12–48 hours post-infection were virtually identical to that of Ad5Luc1, indicating that no significant growth defect is present. To confirm the thermostability of Ad5Luc1-PK virus particles, we performed viral capsid thermostability assays [37]. Equal quantities of Ad5Luc1 or Ad5Luc1-PK (10^10^ v.p.) were incubated at −80°, 4° and 37°C for 3 or 6 days followed by titration of vectors in triplicate by TCID_50_ assay on HEK 293 cells to quantify remaining vector infectivity. Both vectors displayed similar infectious titers at all temperatures and time points, confirming that the presence of the chimeric fiber in Ad5Luc1-PK virions does not alter vector thermostability (data not shown).

### AdLuc1-PK provides enhanced gene delivery

**Figure 3:**
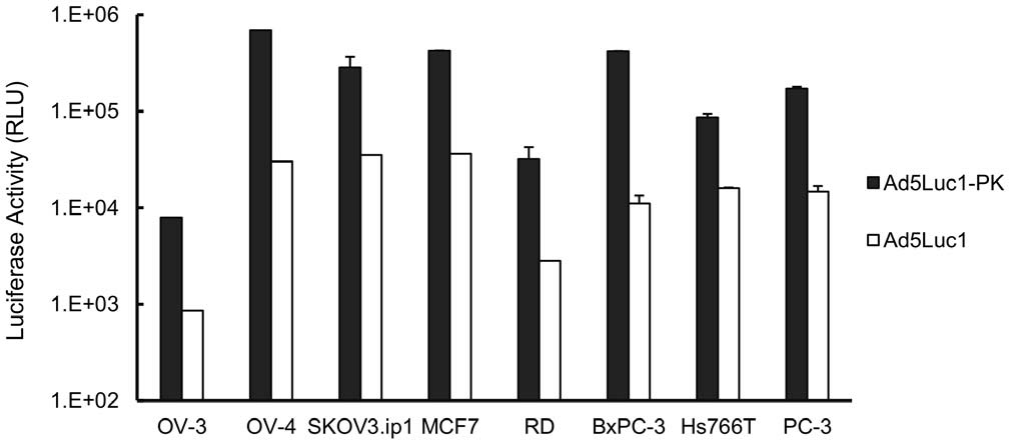
Ad5Luc1-PK vector provides augmented gene transfer. Luciferase activities following infection of cancer cell lines with Ad5Luc1-PK (filled columns) and Ad5Luc1 (open columns). Luciferase activity was determined 24 hours post-infection and reported in relative light units, RLU. Each column is the average of 4 independent replicates using 100 v.p./cell. Error bars indicate standard deviation.

We reasoned that Ad5Luc1-PK would provide augmented transduction through expanded cellular tropism that does not require CAR. We therefore compared Ad5Luc1-PK and Ad5Luc1 transduction in a panel of cancer cell lines from several tissue types which express low levels of CAR [38], [39]. Ad5Luc1-PK provided increased reporter gene delivery to all cell lines compared to Ad5Luc1 (Fig. 3), with augmentation ranging from 8-fold to 23-fold in three ovarian cancer cells lines (OV-3, OV-4 and SKOV3.ip1) and 5-fold to 37-fold in pancreatic carcinoma cell lines HS766T and BxPC-3. Gene transfer in all other cell lines was increased by at least 10-fold compared to Ad5Luc1.

### Ad5-PK vector infectivity is independent of CAR

**Figure 4:**
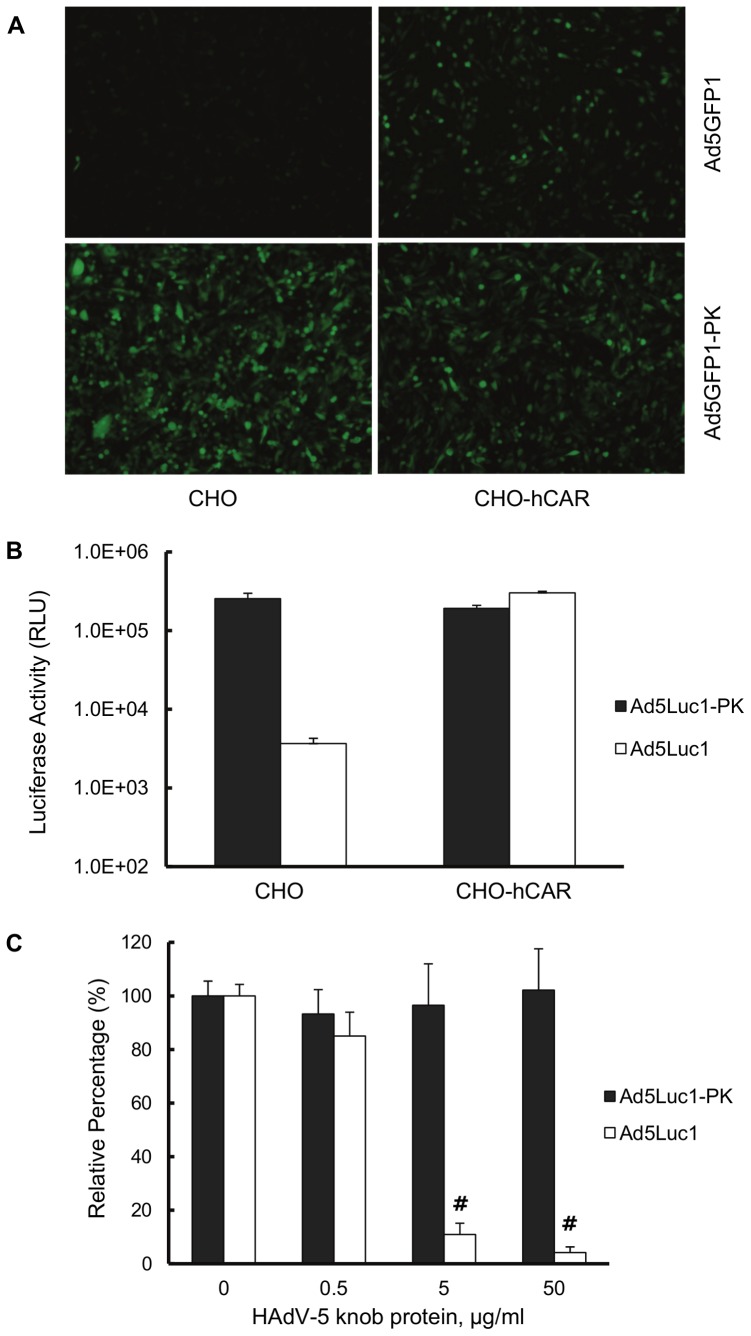
Gene transfer of Ad5-PK vectors is CAR-independent. (A) Fluorescence micrographs of CAR-negative CHO and human CAR (hCAR)-positive CHO-hCAR cell lines imaged 24 hours post-infection with Ad5GFP1-PK (300 v.p./cell), a vector that is isogenic to Ad5Luc1-PK except for the EGFP reporter gene. (B) Luciferase activities in CAR-negative CHO and CAR-positive CHO-hCAR cells following infection with Ad5Luc1 (open columns) or Ad5Luc1-PK (filled columns). Luciferase activities were determined 24 hours post-infection and reported in relative light units, RLU. Each column is the average of 3 independent replicates using 300 v.p./cell. Error bars indicate standard deviation. (C) Luciferase activities in CHO-hCAR cells following infection with Ad5Luc1 (open columns) or Ad5Luc1-PK (filled columns) in the presence of increasing concentrations of recombinant HAdV-5 fiber knob protein to competitively inhibit CAR-mediated cell binding of Ad vectors. Luciferase activities were determined 24 hours post-infection and reported in relative light units, RLU. Each column is the average of 4 independent replicates using 100 v.p./cell. Error bars indicate standard deviation. # indicates that p<0.0002 versus unblocked Ad5Luc1 control using the Student's *t*-test.

High resolution crystal structure analysis has shown that the A-B loop in the N-terminal region of the PAdV-4 knob domain is structurally similar to the A-B loop in the CAR-binding domain in adenoviruses that use CAR as a primary receptor, but that only one CAR-binding residue is conserved [21]. To investigate whether Ad5-PK vectors exhibit CAR-dependent tropism, we performed gene transfer assays in two cell lines with markedly different levels of CAR expression: CAR-deficient Chinese hamster ovary (CHO) cells and a CHO-derived cell line, CHO-hCAR, which stably expresses human CAR (hCAR) [5]. We infected these CHO and CHO-hCAR cells with the Ad5GFP1 control vector and Ad5GFP1-PK, a vector isogenic to Ad5Luc1-PK except that the firefly luciferase reporter gene was replaced with green fluorescent protein, GFP. Fluorescence microscopy showed GFP expression in infected CHO-hCAR cells but not in CAR-deficient CHO cells (Fig. 4A), consistent with native HAdV-5 tropism. In contrast, Ad5Luc1-PK-mediated GFP gene delivery does not depend on CAR expression, as similar number of GFP-positive cells were observed in both the CHO and CHO-hCAR cell lines. We next performed similar gene transfer assays using luciferase-expressing Ad5Luc1 and Ad5Luc1-PK vectors to quantify differences in gene delivery based on CAR expression. Ad5Luc1 exhibited the expected CAR-dependent tropism as demonstrated by an 80-fold increase in luciferase activity in CHO-hCAR cells versus CHO cells (Fig. 4B). In contrast, Ad5Luc1-PK provided robust gene delivery to both cell lines. Further, competitive inhibition of CAR binding with recombinant HAdV-5 knob protein (50 µg/ml) blocked over 96% of Ad5Luc1 gene transfer to CHO-hCAR cells, but not gene transfer of Ad5Luc1-PK (Fig. 4C). Taken together, we conclude that Ad5Luc1-PK tropism is CAR-independent, consistent with the aforementioned structural analysis showing the lack of canonical CAR-binding residues in the PAdV-4 knob domain.

### Glycan dependent infection of Ad5Luc1-PK

**Figure 5:**
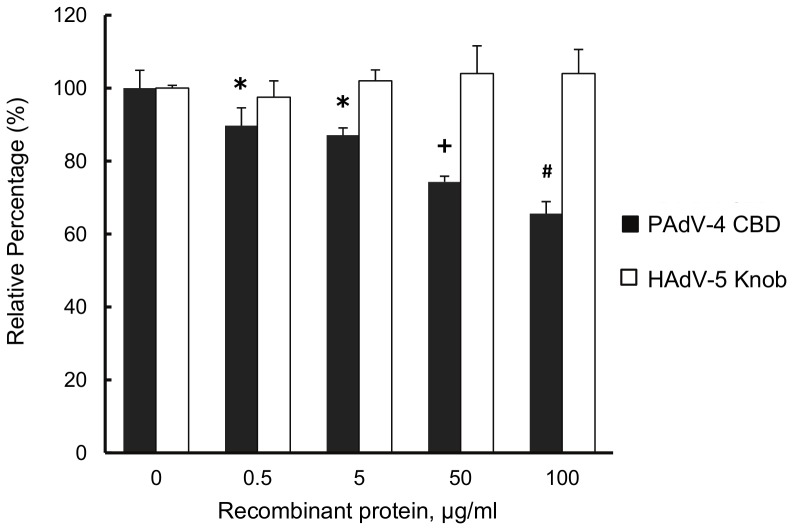
Ad5Luc1-PK uses carbohydrate binding domains for gene transfer. Luciferase activities are expressed as percent of unblocked control groups following infection of BxPC-3 cells with Ad5Luc1-PK in the presence of increasing concentrations of recombinant protein consisting of the PAdV-4 CBD (residues 393–703 of the PAdV-4 fiber protein) (filled columns) or recombinant HAdV-5 fiber knob protein (open columns). Each column is the average of 4 independent replicates using 100 VP/cell. Error bars indicate standard deviation. * indicates p<0.025 versus unblocked control; + indicates p<0.001 versus unblocked control. # indicates that p<0.0002 versus unblocked control using the Student's *t*-test.

To determine whether the CBDs in the chimeric fiber of Ad5Luc1-PK participate in cellular attachment, we performed competitive inhibition assays using a recombinant protein consisting of the tandem PAdV-4 CBDs (residues 393–703 of the PAdV-4 fiber protein) or recombinant HAdV-5 fiber knob protein as a negative control. Addition of PAdV-4 CBD protein during infection caused a dose-dependent inhibition of Ad5Luc1-PK-mediated gene transfer with a maximum inhibition of 35% at 100 µg/ml (Fig. 5), indicating that the CBDs in the chimeric fiber are responsible for cellular attachment during infection.

**Figure 6:**
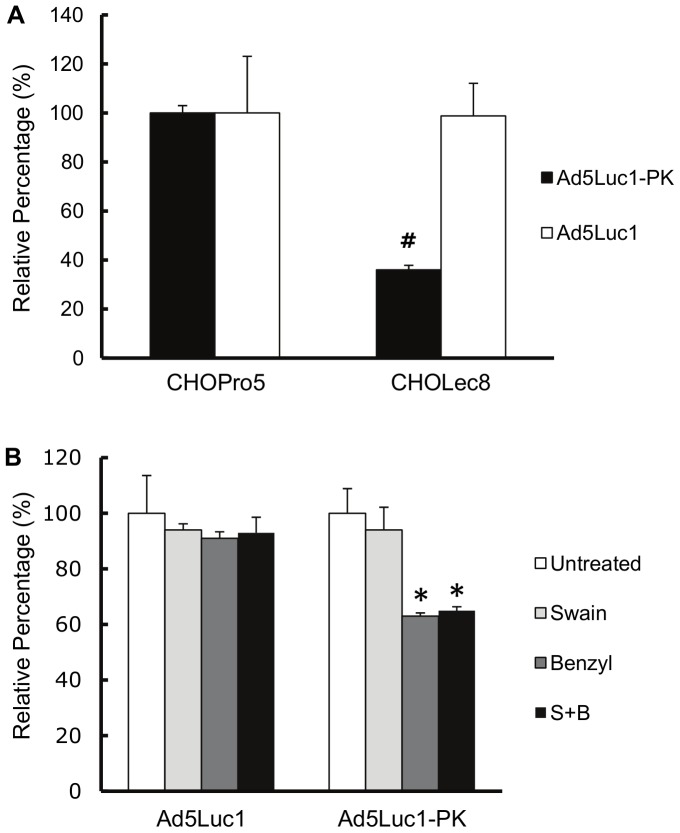
Ad5Luc1-PK-mediated gene delivery is mediated by glycans containing lactose. (A) Luciferase activities expressed as percent of infection of control CHO-Pro5 cells and lactose glycan-deficient CHO-Lec8 cells with Ad5Luc1 (open columns) or Ad5Luc1-PK (filled columns). (B) Luciferase activities expressed as percent of untreated control following infection of CHO-Pro5 cells with Ad5Luc1 or Ad5Luc1-PK with no treatment (open columns), or with 24 hour pre-treatment with the *N*-linked glycosylation inhibitor Swainsonine at a final concentration of 10 µg/ml (light gray columns), with the *O*-linked glycosylation inhibitor benzyl-α-GalNAc at a final concentration of 1 µg/ml (dark gray columns) or with both Swainsonine and benzyl-α-GalNAc (black columns). Each column is the average of 3 independent replicates using 300 v.p./cell. Error bars indicate standard deviation. * indicates p<0.025 versus unblocked control; # indicates that p<0.0002 versus unblocked control using the Student's *t*-test.

The CBDs within the PAdV-4 fiber protein bind to lactose, *N*-actyl-lactosamine and poly-*N*-acetyl-lactosamine in order of increasing affinity [21]. However, whether the CBDs in the PAdV-4 chimeric fiber recognize these glycans and use them as a means for viral transduction is not known. We therefore performed gene transfer assays in CHO-Lec8 cells which contain mutations in the UDP-galactose transporter/translocase (UGT) gene [40], [41], [42]. These cells lack the ability to galactosylate glycoproteins and produce glycoproteins with truncated carbohydrate chains that lack lactose, *N*-acetyl-lactosamine and poly-*N*-acetyl-lactosamine. The level of Ad5Luc1 gene delivery was unchanged between CHO-Lec8 cells and the control CHO-Pro5 cells that exhibit normal glycosylation (Fig. 6A). In contrast, Ad5Luc1-PK gene delivery to CHO-Lec8 cells was reduced by 64% compared to the control CHO-Pro5 cells, confirming that lactose-containing glycans at the cell surface is critical for Ad5Luc1-PK infectivity.

There are two major types of carbohydrate chains on glycoproteins; *N*-linked glycans linked to asparagine residues and *O*-linked glycans linked to serine or threonine [43], [44], [45]. To further investigate the nature of the glycans recognized during Ad5Luc1-PK infection, we performed gene transfer assays following incubation of CHO-Pro5 cells with inhibitors of *N*-linked glycan synthesis (swainsonine, 10 µg/ml) [46], [47], or *O*-linked glycan synthesis (benzyl-α-GalNAc, 1 µg/ml) [46], [47]. The addition of these inhibitors singly or in combination to CHO-Pro5 cells did not alter levels of Ad5Luc1 gene transfer (Fig. 6B). In contrast, Ad5Luc1-PK gene transfer was blocked 35% by benzyl-α-GalNAc pre-treatment, with a minimal (<10%) reduction by swainsonine. We also observed similar results in A549 cells pre-treated with these inhibitors (data not shown), suggesting that *O*-linked cell-surface glycans may be preferred by Ad5Luc1-PK for infection. Collectively, these data show that the CBDs in the chimeric fiber protein of Ad5Luc1-PK directly participate in cellular attachment and that infection is highly dependent on the presence of lactose and/or *N*-acetyl-lactosamine-containing glycans, consistent with a novel, glycan-mediated cell entry pathway.

### Biodistribution of AdLuc1-PK gene expression

**Figure 7:**
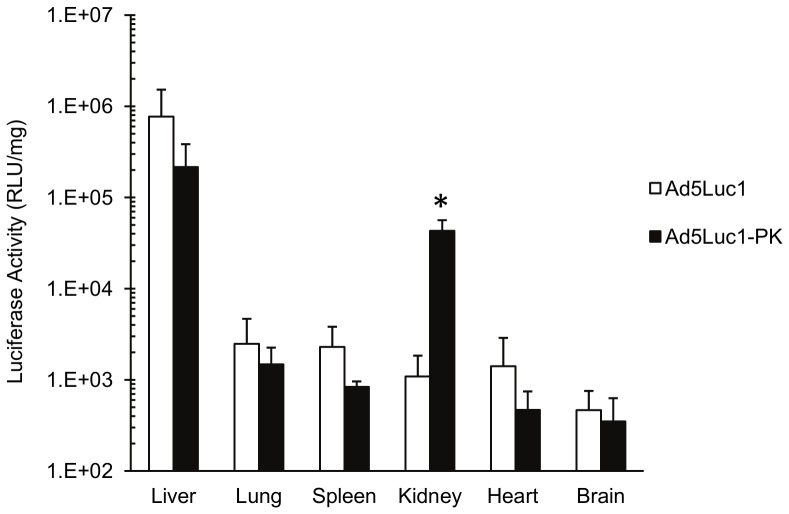
Biodistribution of Ad vector gene expression following intravenous administration. A single dose of 1×10^11^ v.p. of Ad5Luc-1 (open columns) or Ad5Luc1-PK (filled columns) was administered intravenously into the tail vein of C57BL/6 mice. Luciferase activity in tissue lysates was measured 48 hours post-injection and is presented as relative light units (RLU) per milligram of total protein in the lysate. This experiment was performed independently two times with six animals per vector group each time. The results of a single experiment are presented. Error bars indicate standard deviation. * indicates p<0.001 versus Ad5Luc1 using the Student's *t*-test.

It has been shown that structural changes to the fiber protein can alter the biodistribution of systemically administered HAdV vectors [48]. To determine whether the substitution of the PAdV-4 knob domain alters the biodistribution of Ad5Luc1-PK compared to the unmodified control Ad5Luc1 vector, we determined the biodistribution of transgene expression in C57BL/6 mice following intravenous administration. Mice were injected via the tail vein with 1×10^11^ viral particles. Forty-eight hours post-injection the liver, lung, spleen, kidney, heart and brain were harvested and homogenized, and luciferase activity and protein concentrations of cleared tissue homogenates were measured. Ad5Luc1-PK gene expression in the liver, lung heart, spleen, heart and brain was not statistically different from that of Ad5Luc1 (Fig. 7). However, Ad5Luc1-PK gene expression in the kidney was increased approximately 40-fold (p<0.001) compared to the Ad5Luc1 control vector.

## Discussion

Altered glycosylation appears to be a universal hallmark of cancer, and unlike many other cellular dysfunctions that occur throughout tumorogenesis, aberrant glycoconjugates are present on the cell surface and available for direct analysis. On this basis, glycosylation changes represent a major source of cancer-specific biomarkers. Indeed, the serological detection and monitoring of cancer-related carbohydrate antigens is widely used in clinical medicine [18], [49], [50]. To date, however, there has been little progress towards developing therapeutic strategies that target disease-associated carbohydrate species. The objective of this study was to provide initial proof-of-concept of a gene delivery strategy based on vector recognition of specific carbohydrate forms on target cells.

To accomplish this, we generated a novel HAdV-5-based vector, Ad5Luc1-PK, which encodes a chimeric fiber protein containing the tandem carbohydrate binding domains found naturally in the C-terminus of the fiber of PAdV-4, NADC-1 isolate [19]. While the family *Adenoviridae* contains over 100 known serotypes, the presence of carbohydrate binding domains is unique to PAdV-4 and is presumed to have evolved from insertion of a vertebrate gene [51]. In this regard, analysis of the PAdV-4 CBDs has revealed remarkable sequence and structural similarities to the CBDs found in galectins, a family of galactose-binding lectins [21]. There are at least 15 distinct galectins in mammals, each containing one or two conserved CBDs of about 130 amino acids. Members of the galectin family are involved in diverse functions including apoptosis, cellular proliferation, immune responses and cell adhesion and migration [52], [53], [54]. It is therefore not surprising that various galectins have been recently associated with cancer in a variety of tumor types [55], [56], [57]. Galectins are grouped into 3 subtypes (prototype, chimera and tandem repeat) based on the number and intramolecular positioning of the CBDs [58]. Sequence alignment of tandem repeat galectin CBDs has shown that the tandem PAdV-4 CBDs contained in the Ad5-PK vectors are structurally most similar to those of human galectin-9, an observation consistent with the fact that both galectin-9 and Ad5Luc1-PK bind *N*-acetyl-lactosamine and poly-*N*-acetyllactosamine structures [21], [59].

As mentioned above, clinically relevant tissues are often refractory to HAdV-5 vector infection, including many cancer cell types, due to negligible CAR levels. Given that the fiber protein is the primary structural determinant of Ad tropism, we and others have used a genetic fiber pseudotyping approach to replace the HAdV-5 fiber knob domain with the corresponding domain from another Ad serotype that uses a primary receptor other than CAR [2], [25], [35]. This approach allowed the simultaneous elimination of CAR-dependent tropism and addition of the carbohydrate specificity provided by the PAdV-4 CBDs. Despite the 3-fold increase in size (583 amino acids compared to 182 for the HAdV-5 knob) and structural complexity of the PAdV-4 knob domain, western blot analysis confirmed that the fusion of the PAdV-4 knob domain and the HAdV-5 fiber shaft domain results in a fully trimerized, capsid-incorporated chimeric fiber protein. This was a critical outcome, as defects in fiber trimerizarion and/or stability lead to poor capsid incorporation and adversely influence vector rescue, propagation and gene transfer [60].

We characterized the tropism of Ad5Luc1-PK using competitive inhibition assays and well-characterized cell lines with differential expression of CAR or lactose-containing glycoconjugates. We first addressed the question of whether Ad5Luc1-PK maintains the ability to bind CAR, as the AB-loops in the PAdV-4 knob domain are structurally similar to the AB-loops in CAR-binding Ads [21]. Our data clearly show that Ad5-PK vectors do not use CAR for infection, as demonstrated by equivalent gene delivery to cell lines differing in CAR expression as well as a lack of inhibition by recombinant HAdV-5 knob protein at concentrations that block a CAR-dependent vector. These findings are consistent with our previous studies showing Ad5Luc1-PK provides increased gene transfer to CAR-deficient gliomablastoma cell lines [61] as well as to a panel of cancer cell lines of various lineages with low CAR expression (Fig. 3).

While we had previously shown that the PAdV-4 CBDs in recombinant form bind lactose-containing glycans, the function of these CBDs within the structural context of a chimeric fiber and in cell infection was unknown. We first addressed the role of the PAdV-4 knob domain in cell binding via competitive inhibition using purified recombinant PAdV-4 CBD protein. Of note, this protein is identical to that used to generate a high-resolution crystal structure of the CBDs [20]. Our data show that the presence of free excess PAdV-4 CBD protein inhibits Ad5Luc1-PK infection, consistent with the expected role of the chimeric fiber in cell attachment via glycan binding. While the block was significant, it did not reach the levels of inhibition of CAR-dependent vectors commonly observed with HAdV-5 knob at 50–100 µg/mL final concentration (Fig. 4C). We suspect this modest level of blocking is a result of the greatly increased number of glycan binding sites present compared to CAR, and that increased concentrations of PAdV-4 CBD protein would provide more robust block of cell attachment and subsequent gene transfer.

We next confirmed that Ad5Luc1-PK infection depends heavily on the presence of lactose-containing glycans by comparing gene transfer between CHO-Pro5 cells and CHO-Lec8 cells that specifically lack *N*-acetyl-lactosamine and poly-*N*-acetyllactosamine glycans at the cell surface [40], [62]. In addition to the lack of lactose-containing glycans, CHO-Lec8 glycoforms also lack sialic acid (since addition of sialic acid requires a terminal galactose) allowing for the possibility that sialic acid may also be a receptor for Ad5Luc1-PK. HAdV-37 binds sialic acid via interaction with a patch of positively charged residues at the surface of the knob domain [63]. While binding of sialic acid by Ad5Luc1-PK cannot be ruled out without further investigation, the lack of a similar positively charged surface region in the PAdV-4 knob domain makes this possibility unlikely [21].

Systemic administration of HAdV-5 vectors results in significant liver uptake and hepatocyte transduction that can result in liver toxicity and has been a significant impediment to efficient transduction of non-liver target tissues [64], [65], [66]. While this effect is mediated primarily by the interaction of various blood factors with the HAdV-5 hexon protein and hepatocytes [67], [68], it has been shown that structural changes to the HAdV-5 fiber can alter both vector and gene expression biodistribution [39], [48], [69]. In this regard, our data show that biodistribution of Ad5Luc1-PK gene expression following systemic administration is similar to that of Ad5Luc1, save for a trend toward decreased liver expression (p = 0.15) and increased gene expression in the kidney. That Ad5Luc1-PK liver transduction was not significantly different from Ad5Luc1 is not surprising. Both vectors contain two capsid locales implicated in liver transduction *in vivo*, native hexon proteins and the putative heparan sulfate proteoglycan (HSPG)-binding motif, KKTK, in the third repeat of the native HAdV-5 fiber shaft [70]. Our observation of increased kidney gene expression was unexpected, given the relative difficulty of achieving appreciable HAdV-5 gene delivery to the kidney following systemic administration in rodents [71], [72], [73]. Indeed, prolonged exposure to the vector combined with catheter infusion into renal arteries [73], [74], retrograde perfusion systems [75] and direct interstitial injection [76] have been used to increase gene delivery to the rodent kidney using HAdV-5 vectors.

The kidney contains numerous substructures including complex vasculature, glomeruli, tubules and interstitium. While the mechanism of enhanced kidney gene expression remains under investigation, we posit this result to be a consequence of unique interaction(s) of Ad5Luc1-PK with the fenestrated glomerular capillary endothelium and/or the underlying filtration membrane and the epithelium of the renal tubule system.

In conclusion, we have engineered a HAdV-5 vector with a unique carbohydrate binding capacity which provides CAR-independent gene transfer via recognition of cell surface glycans. The ability to target glycoconjugates may offer a promising adjunctive approach to achieve enhanced infectivity for HAdV-5-resistant cellular targets. Further development of this new targeting paradigm may allow vector targeting to specific disease-associated glycan biomarkers or to cell populations that are otherwise refractory to gene delivery.
